# Effects of diabetes mellitus and glycemic traits on cardiovascular morpho-functional phenotypes

**DOI:** 10.1186/s12933-023-02079-w

**Published:** 2023-12-08

**Authors:** Zhaoyue Li, Jie Xiong, Yutong Guo, Hao Tang, Bingchen Guo, Bo Wang, Dianyu Gao, Zengxiang Dong, Yingfeng Tu

**Affiliations:** 1 Harbin Medical University, Harbin, China; 2 Department of Cardiology, the First Affiliated Hospital, Harbin Medical University, Harbin, China; 3 The Key Laboratory of Cardiovascular Disease Acousto-Optic Electromagnetic Diagnosis and Treatment in Heilongjiang Province, The First Affiliated Hospital of Harbin Medical University, Harbin, China; 4 NHC Key Laboratory of Cell Transplantation, The First Affiliated Hospital of Harbin Medical University, Harbin, China

**Keywords:** Diabetes Mellitus, Glycemic traits, Cardiovascular magnetic resonance imaging, Cardiovascular structure and function, Mendelian randomization

## Abstract

**Background:**

The effects of diabetes on the cardiac and aortic structure and function remain unclear. Detecting and intervening these variations early is crucial for the prevention and management of complications. Cardiovascular magnetic resonance imaging-derived traits are established endophenotypes and serve as precise, early-detection, noninvasive clinical risk biomarkers. We conducted a Mendelian randomization (MR) study to examine the association between two types of diabetes, four glycemic traits, and preclinical endophenotypes of cardiac and aortic structure and function.

**Methods:**

Independent genetic variants significantly associated with type 1 diabetes, type 2 diabetes, fasting insulin (FIns), fasting glucose (FGlu), 2 h-glucose post-challenge (2hGlu), and glycated hemoglobin (HbA1c) were selected as instrumental variables. The 96 cardiovascular magnetic resonance imaging traits came from six independent genome-wide association studies. These traits serve as preclinical endophenotypes and offer an early indication of the structure and function of the four cardiac chambers and two aortic sections. The primary analysis was performed using MR with the inverse-variance weighted method. Confirmation was achieved through Steiger filtering and testing to determine the causal direction. Sensitivity analyses were conducted using the weighted median, MR-Egger, and MR-PRESSO methods. Additionally, multivariable MR was used to adjust for potential effects associated with body mass index.

**Results:**

Genetic susceptibility to type 1 diabetes was associated with increased ascending aortic distensibility. Conversely, type 2 diabetes showed a correlation with a reduced diameter and areas of the ascending aorta, as well as decreased distensibility of the descending aorta. Genetically predicted higher levels of FGlu and HbA1c were correlated with a decrease in diameter and areas of the ascending aorta. Furthermore, higher 2hGlu levels predominantly showed association with a reduced diameter of both the ascending and descending aorta. Higher FIns levels corresponded to increased regional myocardial-wall thicknesses at end-diastole, global myocardial-wall thickness at end-diastole, and regional peak circumferential strain of the left ventricle.

**Conclusions:**

This study provides evidence that diabetes and glycemic traits have a causal relationship with cardiac and aortic structural and functional remodeling, highlighting the importance of intensive glucose-lowering for primary prevention of cardiovascular diseases.

**Supplementary Information:**

The online version contains supplementary material available at 10.1186/s12933-023-02079-w.

## Background

Diabetes, a widespread metabolic disease, significantly contributes to cardiovascular-related disability and death, leading to an estimated annual cost of $37.3 billion [1]. Diabetes clearly damages microvessels and macrovessels, but the pathophysiological changes in the heart and aorta, responsible for circulatory pumping, remain unclear. Detecting and intervening these variations early is crucial for prevention and treatment of cardiovascular disease. Several observational studies have tried to associate diabetes and specific glycemic traits with traits like ventricular strain, ejection fraction, and aortic diameter [2–4]. However, these findings are inconsistent and incomplete. Furthermore, these observational studies often face biases due to residual confounding, misclassification, and reverse causation, which challenge the determination of causality.

Cardiac and aortic structures, which underpin physiological functions, can exhibit abnormalities even before overt disease symptoms manifest. Cardiovascular magnetic resonance imaging (CMR) is considered the gold standard for non-invasive evaluation of cardiovascular structure and function [5]. Its derived traits are well-established endophenotypes, serving as risk biomarkers and imaging surrogate endpoints in clinical trials [6, 7]. With the growth of artificial intelligence and the emergence of biobanks, large-scale genome-wide association studies (GWAS) on CMR traits have become possible. These studies have greatly deepened our genetic understanding of the structural and functional remodeling of cardiac and aortic during disease development and progression.

Mendelian randomization (MR), leveraging genetic variations from GWAS analyses as instrumental variables, helps overcome these biases inherent to observational research, thus reinforcing causal connections between exposures and outcomes [8]. Here, we analyzed 96 CMR traits from six GWAS studies, covering four left atrial, four right atrial, 66 left ventricular, four right ventricular, 11 ascending aortic and seven descending aortic traits. Our two-sample MR study aimed to reveal causal relationships between two diabetes types: type 1 and 2 diabetes, and four glycemic traits: fasting insulin (FIns), fasting glucose (FGlu), 2 h-glucose post-challenge (2hGlu) and glycated hemoglobin (HbA1c) with these CMR features. Clarifying these causal relationships is crucial for the evaluation and management of cardiovascular risks in individuals with diabetes and impaired glycemic homeostasis, and it can enhance our deeper understanding of how these factors contribute to cardiovascular disease onset and progression, paving the way for early detection, risk stratification, non-pharmacological and pharmacological treatments, and overall advancement in intervention strategies.

## Methods

### Study design

**Fig. 1:**
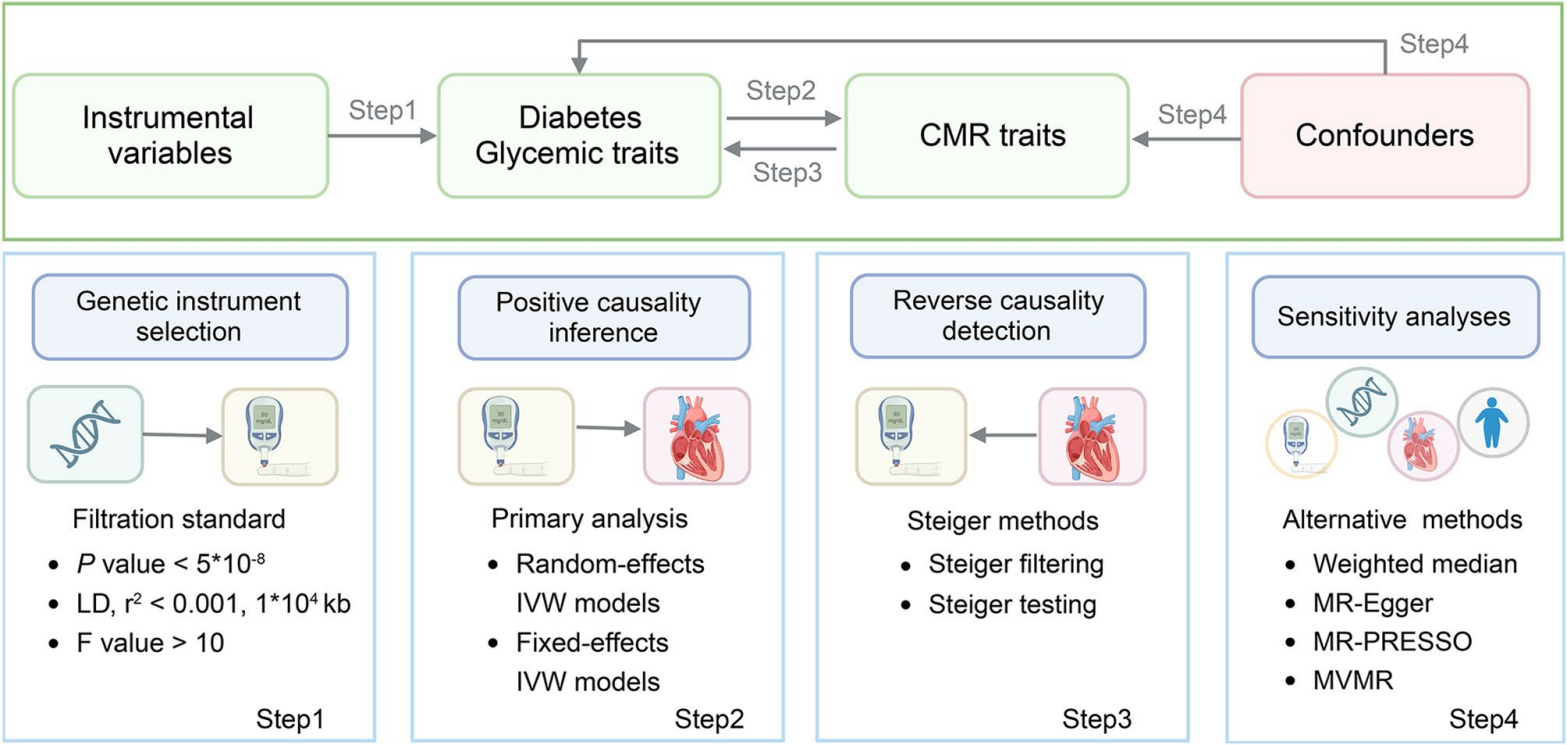
Overview of the study design and analyses. CMR, cardiovascular magnetic resonance; IVW, inverse-variance weighted; LD, linkage disequilibrium; MVMR, multivariable mendelian randomization

This study operated under the three major assumptions of MR analyses: (1) the association assumption, (2) the independence assumption, and (3) the exclusion-restriction assumption. It aimed to explore the individual causal relationships between two types of diabetes and four glycemic traits with 96 CMR traits as endophenotypes, as illustrated in Fig. 1. The summary-level data from GWAS studies used in this research are publicly available, and ethical approvals were obtained in all original papers, as detailed in Additional file 1: Table S1.

### Genetic instrument selection

The genetic instruments associated with type 1 diabetes and type 2 diabetes were derived from studies by Forgetta et al. and the DIAbetes Genetics Replication And Meta-analysis Consortium, respectively [9, 10]. Instruments for the four glycemic traits were sourced from the Meta-Analyses of Glucose and Insulin-related traits Consortium with up to 196,991 individuals of European ancestry without diabetes [11]. To avoid bias from population structure, only European population data were selected from the trans-ancestral datasets related to type 2 diabetes and glycemic traits for exposure data. The criteria for instrumental variable selection were: first, single nucleotide polymorphisms (SNPs) that associated with exposures at the genome-wide significance level (*P* < 5 × 10^− 8^) were selected. Second, LD clumping was performed with a threshold of r^2^ > 0.001 and clump distance < 10,000 kb (1000 genome reference panel). SNPs that displayed linkage disequilibrium were excluded. Subsequently, F-statistic were used to estimate the strength of the genetic instruments. Only genetic instruments with an F-statistic greater than 10 were kept for further analysis to avoid the inclusion of weak instruments. Additional file 1: Tables S2 and S3 contain detailed SNPs information from this study.

### Outcome data sources

This study included 96 CMR features, comprising four left atrial traits, 66 left ventricular traits, four right atrial traits, four right ventricular traits, 11 ascending aortic traits, and seven descending aortic traits. The GWAS summary-level data for these CMR features were primarily derived from six studies based on the UK Biobank, with participant numbers ranging from 29,684 to 43,230 [6, 12–16]. No overlap existed between the glycemic trait data and the UK Biobank sample. For further details on outcome data, such as CMR traits names, categories, participant numbers, and covariates adjusted for in the GWAS analysis model, please refer to Additional file 1: Table S1 and S4.

### Statistical analysis

Before conducting the MR analysis, the exposure and outcome data were harmonized to remove palindromic SNPs. To avoid potential horizontal pleiotropy, SNPs directly related to the outcome data were further removed, and the directionality of the SNPs was tested. Specifically, SNPs that reached the genome-wide significance (*P* < 5 × 10^− 8^) for the outcome were first removed, followed by the use of Steiger filtering to test the directionality of the association of the remaining instrumental variables with the outcome. Any instrumental variable labeled as FALSE by Steiger filtering, indicating that the SNP explained more variance in the outcome than in the exposure, was excluded from the MR analysis. For the MR analysis, IVW was chosen as the primary statistical method. This method assumes that all SNPs are valid instrumental variables, offering the most precise estimates. Notably, considering heterogeneity, its presence was first assessed through Cochran Q analysis. If the *P* value was above 0.05, we used the fixed-effects IVW method, assuming no heterogeneity. Otherwise, the random-effects IVW method was applied. Owing to the susceptibility of the IVW method to horizontal pleiotropy, further sensitivity analyses were conducted using the weighted median method, MR-Egger, and MR-PRESSO. Specifically, the weighted median method assumes that if 50% of the weight from the instrumental variables is valid, a valid causal relationship assessment can still be provided [17]. The MR-Egger regression intercept can be used to evaluate the potential existence of horizontal pleiotropy. This method can still provide causal estimates even if all instrumental variables are invalid, albeit with reduced precision [18]. Although the weighted median and MR-Egger methods have lower statistical power than the IVW method, if all three methods yield consistent results in the same direction, the causal estimates of the primary analysis method are more reliable. Additionally, the MR-PRESSO method was used to identify and correct horizontal pleiotropy introduced by outlier SNPs [19]. For each obtained exposure-outcome pair, the study further tested the direction of the causal relationship through Steiger directionality test to avoid reverse causation. Importantly, to minimize the effects of obesity, multivariable MR (MVMR) analysis was further conducted using body mass index (BMI)-related instrumental variables provided by Pulit et al. [20]. In this analysis, the extended IVW method was the primary analysis method, and potential horizontal pleiotropy was assessed using the MR-Egger intercept. Additional sensitivity analyses were also conducted to reduce the impact of blood pressure on aortic mechanics. Specifically, SNPs associated with hypertension and blood pressure itself that show genome-wide significance (*P* < 5 × 10^− 8^) were identified using the PhenoScanner tool (http://www.phenoscanner.medschl.cam.ac.uk/) among the genetic instruments for diabetes and glycemic traits [21]. After these SNPs were excluded, MR analysis was performed again on the significant findings of the primary analysis, to confirm the initial results.

All analyses were performed using the TwoSampleMR (version 0.5.7) and MendelianRandomization (version 0.9.0) packages in R (version 4.2.2) [17, 22]. The MR estimates were represented by effect value (β) with 95% confidence interval (CI). For the univariate MR analysis, the Bonferroni-corrected *P* value was set at 8.68 × 10^− 5^ (0.05/576). Associations with an original *P* value < 0.05 but not reaching this threshold were considered suggestive. For the MVMR analysis, the *P* value < 0.05 was considered statistically significant.

## Results

### Identifying genetic instruments for Diabetes and glycemic traits

Figure 1 presents a clear schematic representation of the research methodology, while Additional file 1: Table S1 outlines the summary-level data that form the basis for all subsequent analyses. For the genetic prediction of type 1 diabetes, we curated a set of 45 index SNPs. In contrast, 185 index SNPs were carefully chosen for type 2 diabetes (Additional file 1: Table S2). The genetic instruments for FIns, FGlu, 2hGlu, and HbA1c included 38, 68, 14 and 75 index SNPs, respectively (Additional file 1: Table S2). F-statistics ranged from 22.44 to 3136, confirming the robustness of the instrumental variables and reducing the risk of weak-instrument bias, as supported by Additional file 1: Table S3.

### Diabetes and cardiovascular magnetic resonance traits

**Fig. 2:**
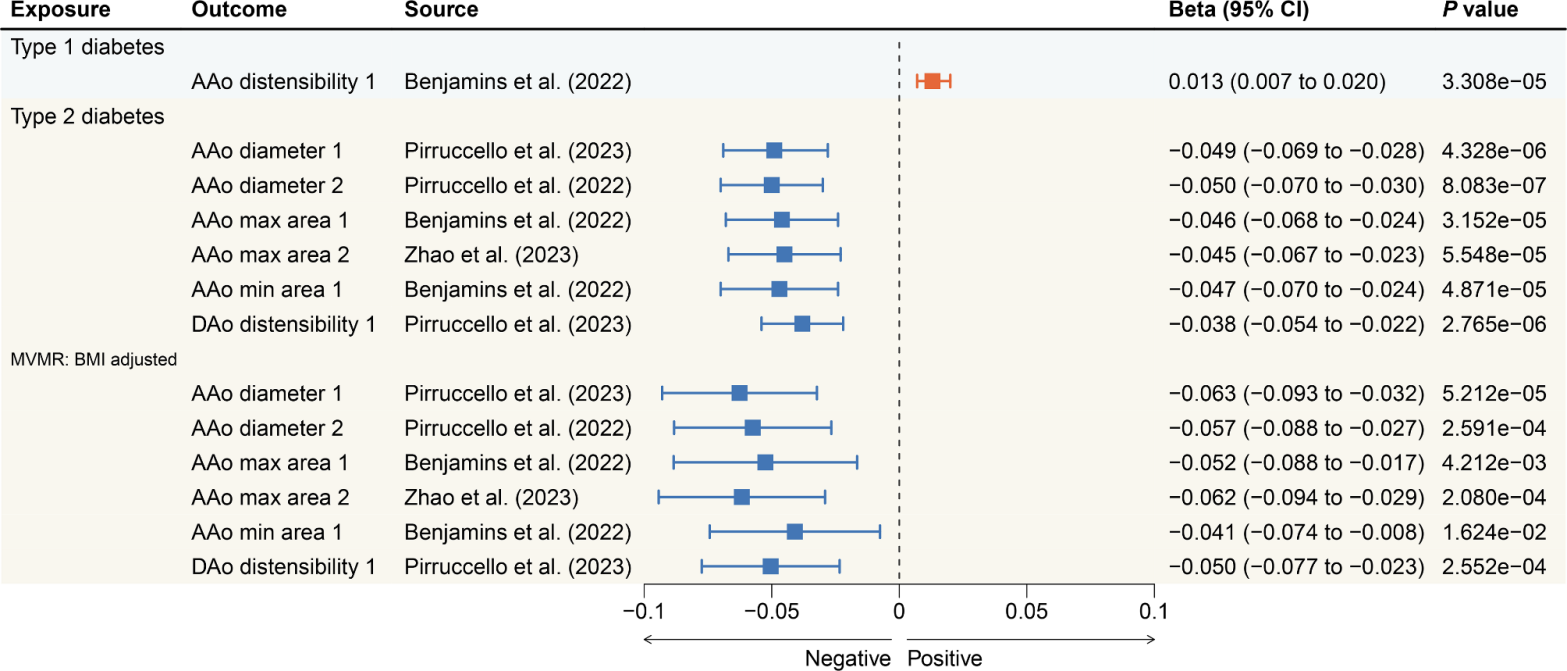
Significant associations of genetic liability to diabetes with cardiovascular magnetic resonance traits. AAo, ascending aorta; DAo, descending aorta; MVMR, multivariable mendelian randomization; BMI, body mass index

In the thorough analysis of all outcome data, a genetic liability for type 1 diabetes showed correlation (*P* < 0.05) with 23 CMR traits (Additional file 1: Table S5). Notably, only the association between type 1 diabetes and increased ascending aortic distensibility (β = 0.013; 95% CI 0.007 to 0.020; *P* = 3.308 × 10^− 5^; Benjamins et al. 2022) passed the stringent criteria of multiple hypothesis testing (Fig. 2). As for type 2 diabetes, its genetic predisposition was associated (*P* < 0.05) with 36 CMR traits (Additional file 1: Table S5). Of these, six CMR traits withstood multiple hypothesis testing (Fig. 2). Specifically, type 2 diabetes was associated with lower ascending aortic diameter (β = -0.049; 95% CI -0.069 to -0.028; *P* = 4.328 × 10^− 6^; Pirruccello et al. 2023 and β = -0.050; 95% CI -0.070 to -0.030; *P* = 8.083 × 10^− 7^; Pirruccello et al. 2022), maximum area (β = -0.046; 95% CI -0.068 to -0.024; *P* = 3.152 × 10^− 5^; Benjamins et al. 2022 and β = -0.045; 95% CI -0.067 to -0.023; *P* = 5.548 × 10^− 5^; Zhao et al. 2023) and minimum area (β = -0.047; 95% CI -0.070 to -0.024; *P* = 4.871 × 10^− 5^; Benjamins et al. 2022). Additionally, type 2 diabetes was also correlated to decreased descending aortic distensibility (β = -0.038; 95% CI -0.054 to -0.022; *P* = 2.765 × 10^− 6^; Pirruccello et al. 2023). To avoid the confounding effects of obesity on type 2 diabetes, a MVMR was used to adjust for BMI. The findings suggested that the relationship between type 2 diabetes and above six CMR traits remains strong following this adjustment (Fig. 2 and Additional file 1: Table S6).

Subsequent sensitivity analyses confirmed the consistent direction of outcomes relative to the genetic susceptibility to both type 1 and type 2 diabetes and their associations with CMR traits (Additional file 1: Tables S7 and S8). Furthermore, no heterogeneity was observed among these statistically significant CMR traits (*P* < 8.68 × 10^− 5^). Additionally, aside from the diameter of the ascending aorta in the context of type 2 diabetes across both studies, no evidence of horizontal pleiotropy was found in the MR-Egger intercept evaluations. The MR-PRESSO assessment identified some outlier SNPs, but their exclusion did not affect the stability of the main findings, thus reinforcing the analytical robustness. Steiger directionality testing was also used to confirm the directional accuracy of the associations between diabetes and CMR traits. All results were considered statistically significant at a *P* value threshold of less than 0.05 (Additional file 1: Table S9). In the sensitivity analysis conducted after removing SNPs related to hypertension and blood pressure, the significant results from the primary analysis remained robust (Additional file 1: Table S10).

### Glycemic traits and cardiovascular magnetic resonance traits

**Fig. 3:**
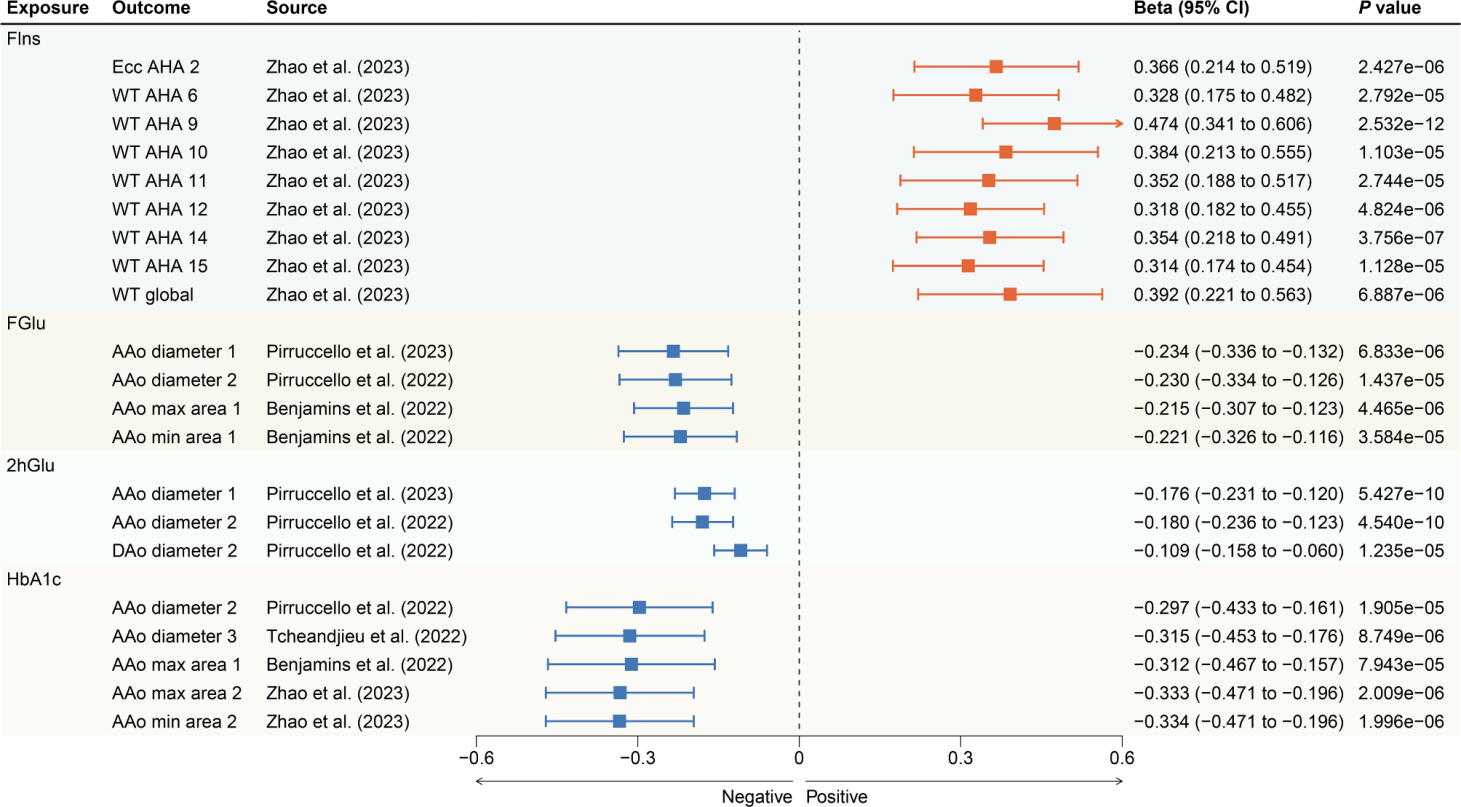
Significant associations of genetically predicted glycemic traits levels with cardiovascular magnetic resonance traits. Ecc AHA, regional peak circumferential strain, segment 2 of the 16 predefined segments by the American Heart Association (AHA); WT AHA, regional myocardial-wall thicknesses at end-diastole, segments 6, 9, 10, 11, 12, 14, and 15 of the 16 predefined segments by the American Heart Association (AHA); WT global, global myocardial-wall thickness at end-diastole; AAo, ascending aorta; DAo, descending aorta; FIns, fasting insulin; FGlu, fasting glucose; 2hGlu, 2 h-glucose post-challenge; HbA1c, glycated hemoglobin

Genetically predicted FIns levels were associated with 37 CMR traits, but only nine passed the multiple hypothesis testing (Additional file 1: Table S5 and Fig. 3). Among these, higher FIns levels were associated with greater regional peak circumferential strain (β = 0.366; 95% CI 0.214 to 0.519; *P* = 2.427 × 10^− 6^; Zhao et al. 2023) (Fig. 3). Furthermore, they were positively correlated with seven regional myocardial-wall thicknesses at end-diastole from the 16 pre-defined American heart association (AHA) segments, as well as with the global myocardial-wall thickness at end-diastole (Fig. 3). The specific values for each are as follows: WT_AHA_6 (β = 0.328; 95% CI 0.175 to 0.482; *P* = 2.792 × 10^− 5^; Zhao et al. 2023), WT_AHA_9 (β = 0.474; 95% CI 0.341 to 0.606; *P* = 2.532 × 10^− 12^; Zhao et al. 2023), WT_AHA_10 (β = 0.384; 95% CI 0.213 to 0.555; *P* = 1.103 × 10^− 5^; Zhao et al. 2023), WT_AHA_11 (β = 0.352; 95% CI 0.188 to 0.517; *P* = 2.744 × 10^− 5^; Zhao et al. 2023), WT_AHA_12 (β = 0.318; 95% CI 0.182 to 0.455; *P* = 4.824 × 10^− 6^; Zhao et al. 2023), WT_AHA_14 (β = 0.354; 95% CI 0.218 to 0.491; *P* = 3.756 × 10^− 7^; Zhao et al. 2023), WT_AHA_15 (β = 0.314; 95% CI 0.174 to 0.454; *P* = 1.128 × 10^− 5^; Zhao et al. 2023) and WT_global (β = 0.392; 95% CI 0.221 to 0.563; *P* = 6.887 × 10^− 6^; Zhao et al. 2023).

FGlu correlated with 25 CMR traits, and 2hGlu with 20 CMR traits (Additional file 1: Table S5). However, after adjusting for multiple hypothesis testing, only four and three traits remained significant, respectively (Fig. 3). Specifically, higher FGlu levels showed a negative association with ascending aortic diameter (β = -0.234; 95% CI -0.336 to -0.132; *P* = 6.833 × 10^− 6^; Pirruccello et al. 2023 and β = -0.230; 95% CI -0.334 to -0.126; *P* = 1.437 × 10^− 5^; Pirruccello et al. 2022), maximum area (β = -0.215; 95% CI -0.307 to -0.123; *P* = 4.465 × 10^− 6^; Benjamins et al. 2022), and minimum area (β = -0.221; 95% CI -0.326 to -0.116; *P* = 3.584 × 10^− 5^; Benjamins et al. 2022). In contrast, higher 2hGlu levels were negatively associated with both the ascending aortic diameter (β = -0.176; 95% CI -0.231 to -0.120; *P* = 5.427 × 10^− 10^; Pirruccello et al. 2023 and β = -0.180; 95% CI -0.236 to -0.123; *P* = 4.540 × 10^− 10^; Pirruccello et al. 2022) and descending aortic diameter (β = -0.109; 95% CI -0.158 to -0.060; *P* = 1.235 × 10^− 5^; Pirruccello et al. 2022).

Genetically predicted HbA1c levels were associated with 24 CMR traits (Additional file 1: Table S5), but after adjusting for multiple hypothesis testing, only five showed a significant negative correlation with HbA1c levels (Fig. 3). These traits included the diameter of the ascending aorta (β = -0.297; 95% CI -0.433 to -0.161; *P* = 1.905 × 10^− 5^; Pirruccello et al. 2022 and β = -0.315; 95% CI -0.453 to -0.176; *P* = 8.749 × 10^− 6^; Tcheandjieu et al. 2022), the maximum area of the ascending aorta (β = -0.312; 95% CI -0.467 to -0.157; *P* = 7.943 × 10^− 5^; Benjamins et al. 2022 and β = -0.333; 95% CI -0.471 to -0.196; *P* = 2.009 × 10^− 6^; Zhao et al. 2023), and the minimum area of the ascending aorta (β = -0.334; 95% CI -0.471 to -0.196; *P* = 1.996 × 10^− 6^; Zhao et al. 2023).

In sensitivity analyses, the associations between glycemic traits and CMR traits remained consistent and were similar to the relationship observed between diabetes and CMR traits. Most results showed no evidence of heterogeneity or horizontal pleiotropy (Additional file 1: Tables S9, S10, S11, S12, S13 and S14).

## Discussion

**Fig. 4:**
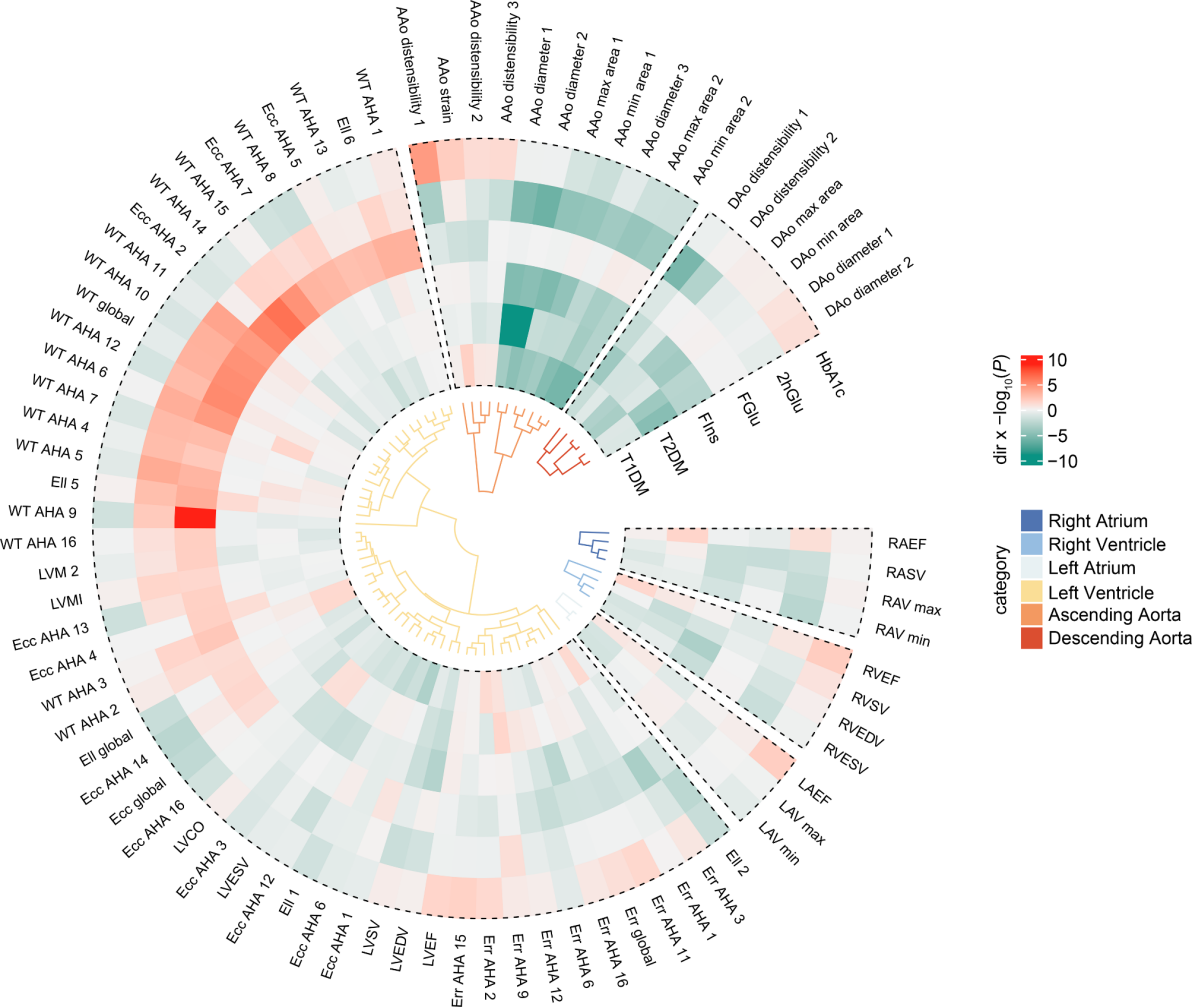
Summary of suggestive and significant associations of genetically predicted diabetes and glycemic traits with cardiovascular magnetic resonance traits. All associations with *P* < 0.05 were clustered based on CMR trait categories and were color-coded according to the effect direction multiplied by the − log10(*P* value). AAo, ascending aorta; DAo, Descending aorta; Ell, longitudinal strain; WT, myocardial-wall thickness at end-diastole; Ecc, circumferential strain; Err, radial strain; LAEF, left atrium ejection fraction; LAV, left atrium volume; LASV, left atrium stroke volume; LVCO, left ventricular cardiac output; LVEF, left ventricular ejection fraction; LVEDV, left ventricular end-diastolic volume; LVESV, left ventricular end-systolic volume; LVM, left ventricular mass; LVMI, left ventricular mass indexed by body surface area; LVSV, left ventricular stroke volume; RAEF, right atrium ejection fraction; RAV, right atrium volume; RASV, right atrium stroke volume; RVEF, right ventricular ejection fraction; RVEDV, right ventricular end-diastolic volume; RVESV, right ventricular end-systolic volume; RVSV, right ventricular stroke volume

The results of this study, which are both suggestive and significant, are summarized in Fig. 4. In this study, we used the latest large-scale GWAS summary-level data to evaluate the relationship of genetically predicted two types of diabetes and four glycemic traits to 96 CMR traits. These CMR features, derived from six GWAS studies, can be divided into six categories, including four left atrial traits, 66 left ventricular traits, four right atrial traits, four right ventricular traits, 11 ascending aortic traits, and seven descending aortic traits [6, 12–16]. To our knowledge, this is the most comprehensive MR study exploring the causal relationship between glucose metabolism and cardiac and aortic structure and function.

**Fig. 5:**
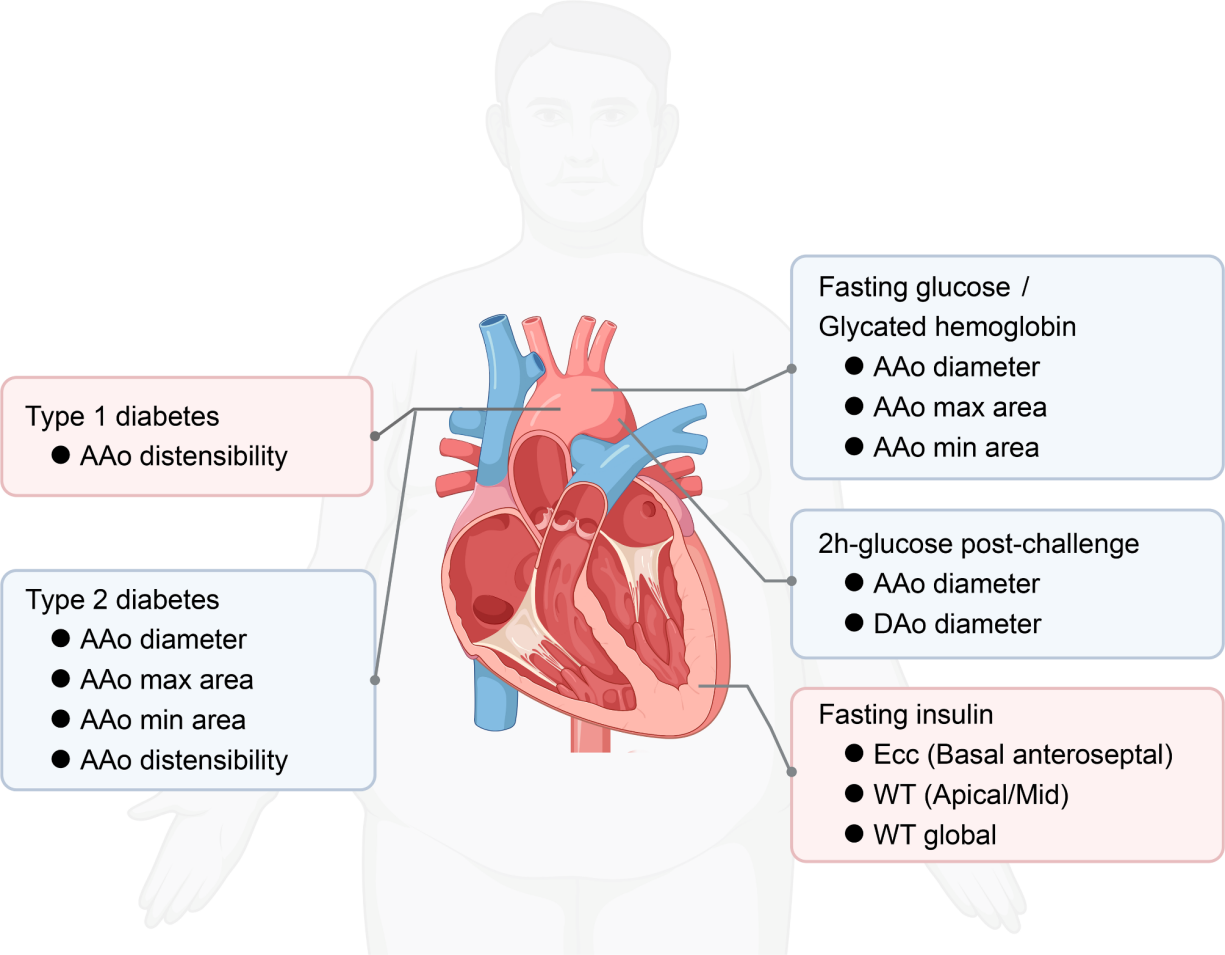
Diagram of the causal association between diabetes, glycemic traits, and the phenotypes of cardiac and aortic structure and function. AAo, ascending aorta; DAo, Descending aorta; Ecc, circumferential strain; WT, myocardial-wall thickness at end-diastole

The primary finding of our study was that diabetes and glycemic traits significantly influence the structural and functional phenotypes of the aorta and the left ventricle (Fig. 5). The results were as follows: (1) Type 1 diabetes was positively correlated with the distensibility of the ascending aorta. (2) Type 2 diabetes was negatively correlated with the diameter, minimum area, and maximum area of the ascending aorta, as well as the distensibility of the descending aorta. (3) FGlu negatively correlated with the diameter, minimum area, and maximum area of the ascending aorta. (4) 2hGlu negatively correlated with the diameters of both the ascending and descending aortas. (5) HbA1c was negative correlated with the diameter, minimum area, and maximum area of the ascending aorta. (6) FIns was positively associated with regional myocardial-wall thicknesses at end-diastole, global myocardial-wall thickness at end-diastole, and regional peak circumferential strain. Examining the relationship between quantitative imaging endophenotypes and modifiable risk factors revealed results with key clinical implications. Diabetes and impaired glucose homeostasis are chronic processes [23]. As a result, harmful cardiac and aortic changes continue and intensify over time. These changes can reach a critical point, leading to a shift from a pre-clinical to a symptomatic cardiovascular disease stage. In addition, these structural and functional changes are also significant from a public health perspective, even on a broader population scale. The reliability and consistency of CMR would make it ideal for monitoring the effects of strong glucose-lowering treatments, helping to assess the effectiveness of primary prevention for cardiovascular disease.

The aorta, the largest elastic artery in the human body, channels blood from the left ventricle [12]. It is associated with several cardiovascular diseases, especially aortic aneurysms, which are significantly influenced by the aortic diameter [24]. Past studies indicated a negative correlation between type 2 diabetes patients and the prevalence and incidence of thoracic and abdominal aortic aneurysms [4, 25, 26]. The aortic root diameter of non-diabetic individuals was typically larger than that of diabetic patients, making them more prone to aortic root expansion [27]. Another observational study revealed a negative association between FGlu levels and the infra-renal aortic diameter [28]. Our current study found that the ascending aortic diameter reduced due to genetically predicted type 2 diabetes and elevated FGlu levels. Various other independent datasets in our research had been also corroborated this conclusion, reinforcing its validity and thus lending genetic support to prior observational claims.

Interestingly, we also newly found that both 2hGlu and HbA1c had the similar effect—reducing the ascending aortic diameter. Moreover, 2hGlu even led to a reduction in the descending aortic diameter based on this observation. FGlu and 2hGlu levels offer insights into short-term glucose regulation, whereas HbA1c reflects the three-month glucose history [29]. Evidently, fluctuations in blood glucose, whether short-term or long-term, can alter the aortic diameter, thereby influencing hemodynamics, an observation that could contribute to further understanding of the negative association between diabetes and aortic aneurysms.

The uncovered relationship between impaired glycemic homeostasis and aortic diameter carries significant clinical implications. The aortic diameter is a predictor of both all-cause mortality and cardiovascular events [30]. Its U-shaped relationship with the risk of cardiovascular death indicates that an overly narrow diameter could have been harmful [31]. This insight underscores the importance of intensive glucose-lowering strategies to manage both short and long-term glycemic levels, aiming to protect against cardiovascular threats.

Besides 2hGlu, we also discovered that type 2 diabetes, FGlu, and HbA1c negatively correlated with the maximum and minimum areas of the ascending aorta. Aortic areas, crucial for the hemodynamics of the circulatory system, are strongly associated with the risk of hypertension [32]. An earlier study showed that genetic increases in these aortic areas heighten aneurysm risk [12]. Our findings suggested that type 2 diabetes and glycemic homeostasis parameters affect not just the aortic diameter but also its area. This might elucidate the unique association of diabetes with aortic disease, distinct from that of hypertension.

The aorta, the largest conduit vessel, can store energy during systole and use it during diastole to ensure blood reaches peripheral vessels. This “windkessel” ability is reflected by the aortic distensibility [14]. Reduced distensibility can hinder blood delivery, strain the heart by increasing ventricular afterload, and predict cardiovascular events and mortality [33]. In our study, type 1 and type 2 diabetes showed distinctly different effects on aortic distensibility. Type 1 diabetes was associated with greater distensibility in the ascending aorta, while type 2 diabetes was linked with decreased distensibility in the descending aorta. Sensitivity analyses suggested that these results are unlikely to be influenced by blood pressure as a confounding factor. Previous research has shown that variants associated with increased expression of TGF-β pathway inhibitors are related to increased aortic distensibility, while variants associated with increased TGF-β signaling are related to decreased aortic distensibility [34]. Upregulation of TGF-β signaling has been found in several diseases with reduced aortic distensibility [35, 36]. Interestingly, serum TGF-β levels are reduced in T1DM patients with autoimmune dysregulation, whereas they are increased in T2DM patients [37–39]. Considering diabetes’ complexity and the different pathophysiological mechanisms between its types, these variations might explain the discrepancies observed. It could suggest that future studies should differentiate between type 1 and type 2 diabetes patients to better understand their relationship with cardiovascular structure and function, and disease onset and progression.

Our study examined both ascending and descending aortic CMR traits. Interestingly, the ascending aorta seems more influenced by diabetes and glycemic traits than the descending aorta. Previous research indicated that the vascular smooth muscle cells of the thoracic ascending aorta have a distinct embryonic origin from the rest of the arterial tree, arising from neural crest cells [40]. Given that, we theorize that the ascending thoracic aorta may be more susceptible to diabetes and disruptions in glycemic balance. While the exact mechanisms connecting impaired glycemic homeostasis to aortic structure and function remain unclear, recent findings pointed to factors such as inflammation, glycation, the TGF-β signaling pathway, extracellular matrix remodelling, and vascular smooth muscle stability [41–45]. However, many insights come from animal studies focusing on the abdominal aorta and may overlook variations between aortic segments. Future research should delve into specific aortic sections, such as the ascending thoracic aorta, aortic arch, and descending thoracic and abdominal aortas, to deepen our understanding of how glycemic homeostasis relates to aortic structure and related diseases.

Diabetic patients have a high prevalence of heart failure, termed diabetic cardiomyopathy, with rates between 19% and 26% [46]. This disease is often asymptomatic in early stages, first showing as a thickening of the left ventricular wall, causing isolated diastolic dysfunction [47, 48]. Our study revealed that higher FIns levels, when genetically predicted, could notably increase the thickness of myocardial-wall thickness at end-diastole and regional peak circumferential strain, leading to changes in left ventricular characteristics, echoing prior studies [49–51]. Interestingly, we found that FIns primarily affected the middle and apex of left ventricle in 16 pre-defined AHA segments [52], including basal anterolateral, mid inferoseptal, mid inferior, mid inferolateral, mid anterolateral, apical septal, and apical inferior. Expanding on prior studies that have already elucidated how fasting insulin affects the geometric shape of the left ventricle (Additional file 1: Tables S15), particularly in terms of increasing its mass and thickness, our research further deepens understanding by uncovering the heterogeneous effects of fasting insulin on different spatial locations within the left ventricle. Early research indicates that cardiomyocytes, primary targets of insulin, suffer impaired glucose utilization in hyperinsulinemic conditions, resulting in an energy-starved state and toxicity [53]. Recent advancements in cardiac single-cell atlas studies have also uncovered significant internal heterogeneity within cardiomyocytes, challenging the prior notion of their homogenesis. These diverse subgroups exhibit distinct transcriptional activities and spatial patterns [54, 55]. Hence, we hypothesize that this heterogeneity might influence their insulin sensitivity, potentially accounting for the uneven impact of insulin on ventricular structure. Further validation of this hypothesis in future research is warranted. In addition, we did not find a significant causal relationship with FGlu, 2hGlu, or HbA1c. This aligns with two recent meta-analyses from randomized controlled trials. They suggested that stricter glucose control does not necessarily lower heart failure risk in type 2 diabetes patients or those at risk [56, 57]. Yet, observational studies showed that with each 1% rise in HbA1c, there is a 30% and 8% increase in heart failure risk for type 1 and type 2 diabetes, respectively [58, 59]. These conflicting findings could be attributed to the unavoidable confounding factors in observational studies. Considering MR analysis can substantially overcome such confounding bias and strengthen causal inference, our findings also suggested that FIns, independent of hyperglycemia, promoted left ventricular remodeling, hastening diabetic cardiomyopathy. It’s worth highlighting that FIns represents both insulin secretion and resistance, and frequently serves as a surrogate marker for insulin resistance in extensive studies [11]. In conclusion, this further provides two main insights: Firstly, hyperglycemia might contribute to cardiovascular disease through other mechanisms, like influencing the structure and function of the aforementioned ascending aorta, which warrants further investigation in both basic and clinical research. Secondly, drugs that can lower FIns and enhance insulin sensitivity, such as sodium-glucose cotransporter 2 inhibitors and glucagon-like peptide-1 receptor agonists, should be prioritized in intensive glucose-lowering therapy to mitigate adverse ventricular remodeling and its associated risk of heart failure [60, 61].

In summary, our study offers several strengths: (1) Through MR analysis, we have shed new light on the causal links between two diabetes types, four glucose traits, and 96 CMR traits spanning six anatomical areas, including the left atrium, right atrium, left ventricle, right ventricle, ascending aorta, and descending aorta. (2) MR study design reduced residual confounding, yielding more robust causal evidence. (3) While reverse causation might skew MR findings, we addressed this by filtering SNPs closely tied to outcomes with the Steiger method. The Steiger directionality test further confirmed our causal assumptions. (4) We compiled GWAS data on multiple CMR traits and validated the relationships between exposures and outcomes in diverse independent datasets. This approach not only highlighted neglected associations but also lend more weight to our causal conclusions given consistent results. (5) Since our findings mainly draw from summary-level data of European individuals, the potential for bias due to population structure is minimal.

This study has several limitations. Firstly, while MR studies ideally should fully avoid horizontal pleiotropy, SNPs related to type 2 diabetes and glycemic traits might also be linked to metabolic factors like obesity. Given that the GWAS data for glycemic traits was adjusted for BMI, making the instrumental variables used likely independent of this factor, thus the impact of obesity-induced pleiotropy was expected to be minimal [11]. For type 2 diabetes, results adjusted for BMI in MVMR analysis aligned with the univariable analysis. In the sensitivity analysis, some associations lost statistical significance. However, even with varying assumptions, the direction of these results aligned with the IVW method. This consistency further underscores the reliability of our findings and indicates that the effects of horizontal pleiotropy on our results are minimal. Second, focusing on glycemic traits from non-diabetic individuals could introduce collider bias. It is unclear whether these traits truly represent glucose metabolic levels in diabetic patients. However, our study found similar associations for both diabetic and non-diabetic patients, warranting further examination in future research. Fourth, using public summary-level data poses challenges in ensuring non-overlapping samples. Yet, all F-statistics in our analyses exceed 10, indicating robust genetic instruments [62]. This suggested that the potential for bias influencing the outcomes is minimal [62]. Fifth, limited by the nature of MR studies focusing on the lifetime exposure effects of traits of interest, they are unable to consider the time-varying effects associated with exposure phenotypes. Novel methods in addressing the time-varying effects of these phenotypes on CMR traits should be examined in the future using datasets with available data. Sixth, since we used summary-level data, the study could not delve into potential non-linear associations, which should be explored in future research. Finally, our primary data sources involved European ancestry individuals, so the findings might not generalize to other ethnicities.

## Conclusions

In summary, we delved into the impact of two diabetes types and four glucose traits on heart and aortic remodeling. Our research shows that both diabetes and hyperglycemia are risk factors for aortic remodeling, whereas insulin resistance affects the left ventricle. These insights, by providing causal evidence of direct effects on cardiac and aortic morphology and function, advance our understanding of how diabetes and impaired glycemic homeostasis contribute to the onset and progression of cardiovascular diseases. It offers new insights for early detection, risk stratification, non-pharmacological and pharmacological treatments, and overall advancement in intervention strategies.

### Electronic supplementary material

Below is the link to the electronic supplementary material.

Supplementary Material 1

## Data Availability

All data generated or analyzed during this study are included in this article and its supplementary information files.

## Abbreviations

AHA: American heart association
BMI: Body mass index
CI: Confidence interval
CMR: Cardiovascular magnetic resonance imaging
GWAS: Genome-wide association study
FIns: Fasting insulin
FGlu: Fasting glucose
2hGlu: 2 h-glucose post-challenge
HbA1c: Glycated hemoglobin
IVW: Inverse-variance weighted
MR: Mendelian randomization
MVMR: Multivariable Mendelian randomization
SNPs: Single nucleotide polymorphisms

## References

[1] American Diabetes A (2018). Economic costs of Diabetes in the U.S. in 2017. Diabetes Care.

[2] Linssen PBC, Veugen MGJ, Henry RMA, van der Kallen CJH, Kroon AA, Schram MT, Brunner-La Rocca HP, Stehouwer CDA (2020). Associations of (pre)Diabetes with right ventricular and atrial structure and function: the Maastricht Study. Cardiovasc Diabetol.

[3] Shi R, Yang ZG, Guo YK, Qian WL, Gao Y, Li XM, Jiang L, Xu HY, Li Y (2023). The right ventricular dysfunction and ventricular interdependence in patients with DM: assessment using cardiac MR feature tracking. Cardiovasc Diabetol.

[4] Ning X, Ding N, Ballew SH, Hicks CW, Coresh J, Selvin E, Pankow J, Tang W, Matsushita K (2020). Diabetes, its duration, and the long-term risk of abdominal aortic Aneurysm: the Atherosclerosis risk in communities (ARIC) Study. Atherosclerosis.

[5] Demirkiran A, Everaars H, Amier RP, Beijnink C, Bom MJ, Gotte MJW, van Loon RB, Selder JL, van Rossum AC, Nijveldt R (2019). Cardiovascular magnetic resonance techniques for tissue characterization after acute myocardial injury. Eur Heart J Cardiovasc Imaging.

[6] Zhao B, Li T, Fan Z, Yang Y, Shu J, Yang X, Wang X, Luo T, Tang J, Xiong D (2023). Heart-brain connections: phenotypic and genetic insights from magnetic resonance images. Science.

[7] Benz DC, Grani C, Antiochos P, Heydari B, Gissler MC, Ge Y, Cuddy SAM, Dorbala S, Kwong RY. Cardiac magnetic resonance biomarkers as surrogate endpoints in cardiovascular trials for myocardial Diseases. Eur Heart J 2023. 10.1093/eurheartj/ehad510 PMC11032206 37700499

[8] Skrivankova VW, Richmond RC, Woolf BAR, Yarmolinsky J, Davies NM, Swanson SA, VanderWeele TJ, Higgins JPT, Timpson NJ, Dimou N (2021). Strengthening the reporting of Observational studies in Epidemiology using mendelian randomization: the STROBE-MR Statement. JAMA.

[9] Forgetta V, Manousaki D, Istomine R, Ross S, Tessier MC, Marchand L, Li M, Qu HQ, Bradfield JP, Grant SFA (2020). Rare genetic variants of large effect influence risk of type 1 Diabetes. Diabetes.

[10] Mahajan A, Spracklen CN, Zhang W, Ng MCY, Petty LE, Kitajima H, Yu GZ, Rueger S, Speidel L, Kim YJ (2022). Multi-ancestry genetic study of type 2 Diabetes highlights the power of diverse populations for discovery and translation. Nat Genet.

[11] Chen J, Spracklen CN, Marenne G, Varshney A, Corbin LJ, Luan J, Willems SM, Wu Y, Zhang X, Horikoshi M (2021). The trans-ancestral genomic architecture of glycemic traits. Nat Genet.

[12] Benjamins JW, Yeung MW, van de Vegte YJ, Said MA, van der Linden T, Ties D, Juarez-Orozco LE, Verweij N, van der Harst P (2022). Genomic insights in ascending aortic size and distensibility. EBioMedicine.

[13] Pirruccello JP, Chaffin MD, Chou EL, Fleming SJ, Lin H, Nekoui M, Khurshid S, Friedman SF, Bick AG, Arduini A (2022). Deep learning enables genetic analysis of the human thoracic aorta. Nat Genet.

[14] Pirruccello JP, Ramo JT, Choi SH, Chaffin MD, Kany S, Nekoui M, Chou EL, Jurgens SJ, Friedman SF, Juric D (2023). The genetic determinants of aortic distention. J Am Coll Cardiol.

[15] Tcheandjieu C, Xiao K, Tejeda H, Lynch JA, Ruotsalainen S, Bellomo T, Palnati M, Judy R, Klarin D, Kember RL (2022). High heritability of ascending aortic diameter and trans-ancestry prediction of thoracic aortic Disease. Nat Genet.

[16] Khurshid S, Lazarte J, Pirruccello JP, Weng LC, Choi SH, Hall AW, Wang X, Friedman SF, Nauffal V, Biddinger KJ (2023). Clinical and genetic associations of deep learning-derived cardiac magnetic resonance-based left ventricular mass. Nat Commun.

[17] Yavorska OO, Burgess S (2017). MendelianRandomization: an R package for performing mendelian randomization analyses using summarized data. Int J Epidemiol.

[18] Burgess S, Thompson SG (2017). Interpreting findings from mendelian randomization using the MR-Egger method. Eur J Epidemiol.

[19] Verbanck M, Chen CY, Neale B, Do R (2018). Detection of widespread horizontal pleiotropy in causal relationships inferred from mendelian randomization between complex traits and Diseases. Nat Genet.

[20] Pulit SL, Stoneman C, Morris AP, Wood AR, Glastonbury CA, Tyrrell J, Yengo L, Ferreira T, Marouli E, Ji Y (2019). Meta-analysis of genome-wide association studies for body fat distribution in 694 649 individuals of European ancestry. Hum Mol Genet.

[21] Kamat MA, Blackshaw JA, Young R, Surendran P, Burgess S, Danesh J, Butterworth AS, Staley JR (2019). PhenoScanner V2: an expanded tool for searching human genotype-phenotype associations. Bioinformatics.

[22] Hemani G, Zheng J, Elsworth B, Wade KH, Haberland V, Baird D, Laurin C, Burgess S, Bowden J, Langdon R et al. The MR-Base platform supports systematic causal inference across the human phenome. Elife 2018, 7. 10.7554/eLife.34408 PMC5976434 29846171

[23] Schiborn C, Schulze MB (2022). Precision prognostics for the development of Complications in Diabetes. Diabetologia.

[24] Girardi LN, Lau C, Gambardella I (2021). Aortic dimensions as predictors of adverse events. J Thorac Cardiovasc Surg.

[25] Takagi H, Umemoto T, Group A (2017). Negative Association of Diabetes with thoracic Aortic Dissection and Aneurysm. Angiology.

[26] Shah AD, Langenberg C, Rapsomaniki E, Denaxas S, Pujades-Rodriguez M, Gale CP, Deanfield J, Smeeth L, Timmis A, Hemingway H (2015). Type 2 Diabetes and incidence of Cardiovascular Diseases: a cohort study in 1.9 million people. Lancet Diabetes Endocrinol.

[27] Nardi E, Mule G, Nardi C, Geraci G, Averna M (2017). Inverse association between type 2 Diabetes and aortic root dimension in hypertensive patients. Int J Cardiol.

[28] Le MT, Jamrozik K, Davis TM, Norman PE (2007). Negative association between infra-renal aortic diameter and glycaemia: the Health in men Study. Eur J Vasc Endovasc Surg.

[29] ElSayed NA, Aleppo G, Aroda VR, Bannuru RR, Brown FM, Bruemmer D, Collins BS, Hilliard ME, Isaacs D, Johnson EL (2023). 2. Classification and diagnosis of Diabetes: standards of Care in Diabetes-2023. Diabetes Care.

[30] Kamimura D, Suzuki T, Musani SK, Hall ME, Samdarshi TE, Correa A, Fox ER. Increased proximal aortic diameter is Associated with Risk of Cardiovascular events and all-cause Mortality in blacks the Jackson Heart Study. J Am Heart Assoc 2017, 6(6). 10.1161/JAHA.116.005005 PMC5669152 28637775

[31] Sidloff DA, Saratzis A, Thompson J, Katsogridakis E, Bown MJ: ‘s Choice - Infra-Renal Aortic Diameter and Cardiovascular Risk, editors. Making Better Use of Abdominal Aortic Aneurysm Screening Outcomes. *Eur J Vasc Endovasc Surg* 2021, 62(1):38–45. 10.1016/j.ejvs.2021.03.013 33985908

[32] Bai W, Suzuki H, Huang J, Francis C, Wang S, Tarroni G, Guitton F, Aung N, Fung K, Petersen SE (2020). A population-based phenome-wide association study of cardiac and aortic structure and function. Nat Med.

[33] Redheuil A, Wu CO, Kachenoura N, Ohyama Y, Yan RT, Bertoni AG, Hundley GW, Duprez DA, Jacobs DR, Daniels LB (2014). Proximal aortic distensibility is an Independent predictor of all-cause mortality and incident CV events: the MESA study. J Am Coll Cardiol.

[34] Francis CM, Futschik ME, Huang J, Bai W, Sargurupremraj M, Teumer A, Breteler MMB, Petretto E, Ho ASR, Amouyel P (2022). Genome-wide associations of aortic distensibility suggest causality for aortic aneurysms and brain white matter hyperintensities. Nat Commun.

[35] Nollen GJ, Groenink M, Tijssen JG, Van Der Wall EE, Mulder BJ (2004). Aortic stiffness and diameter predict Progressive aortic dilatation in patients with Marfan Syndrome. Eur Heart J.

[36] Gallo EM, Loch DC, Habashi JP, Calderon JF, Chen Y, Bedja D, van Erp C, Gerber EE, Parker SJ, Sauls K (2014). Angiotensin II-dependent TGF-beta signaling contributes to Loeys-Dietz syndrome vascular pathogenesis. J Clin Invest.

[37] Qiao YC, Shen J, Hong XZ, Liang L, Bo CS, Sui Y, Zhao HL (2016). Changes of regulatory T cells, transforming growth factor-beta and interleukin-10 in patients with type 1 Diabetes Mellitus: a systematic review and meta-analysis. Clin Immunol.

[38] Qiao YC, Shen J, He L, Hong XZ, Tian F, Pan YH, Liang L, Zhang XX, Zhao HL. Changes of Regulatory T Cells and of Proinflammatory and Immunosuppressive Cytokines in Patients with Type 2 Diabetes Mellitus: A Systematic Review and Meta-Analysis. *J Diabetes Res* 2016, 2016:3694957. 10.1155/2016/3694957 PMC5061980 27777959

[39] Hussain G, Rizvi SA, Singhal S, Zubair M, Ahmad J (2016). Serum levels of TGF-beta1 in patients of diabetic peripheral neuropathy and its correlation with nerve conduction velocity in type 2 Diabetes Mellitus. Diabetes Metab Syndr.

[40] Klarin D, Devineni P, Sendamarai AK, Angueira AR, Graham SE, Shen YH, Levin MG, Pirruccello JP, Surakka I, Karnam PR (2023). Genome-wide association study of thoracic aortic Aneurysm and dissection in the million veteran program. Nat Genet.

[41] Raffort J, Lareyre F, Clement M, Hassen-Khodja R, Chinetti G, Mallat Z (2018). Diabetes and aortic Aneurysm: current state of the art. Cardiovasc Res.

[42] Arapoglou V, Kondi-Pafiti A, Rizos D, Carvounis E, Frangou-Plemenou M, Kotsis T, Katsenis K (2010). The influence of Diabetes on degree of abdominal aortic Aneurysm tissue inflammation. Vasc Endovascular Surg.

[43] Koole D, van Herwaarden JA, Schalkwijk CG, Lafeber F, Vink A, Smeets MB, Pasterkamp G, Moll FL (2017). A potential role for glycated cross-links in abdominal aortic Aneurysm Disease. J Vasc Surg.

[44] Mallat Z, Ait-Oufella H, Tedgui A (2017). The pathogenic transforming growth factor-beta overdrive hypothesis in aortic aneurysms and dissections: a Mirage?. Circ Res.

[45] Cikach FS, Koch CD, Mead TJ, Galatioto J, Willard BB, Emerton KB, Eagleton MJ, Blackstone EH, Ramirez F, Roselli EE et al. Massive aggrecan and versican accumulation in thoracic aortic Aneurysm and dissection. JCI Insight 2018, 3(5). 10.1172/jci.insight.97167 PMC5922288 29515038

[46] Dillmann WH (2019). Diabetic Cardiomyopathy. Circ Res.

[47] Lezoualc’h F, Badimon L, Baker H, Bernard M, Czibik G, de Boer RA, D’Humieres T, Kergoat M, Kowala M, Rieusset J (2023). Diabetic cardiomyopathy: the need for adjusting experimental models to meet clinical reality. Cardiovasc Res.

[48] Ritterhoff J, Tian R. Metabolic mechanisms in physiological and pathological cardiac hypertrophy: new paradigms and challenges. Nat Rev Cardiol 2023. 10.1038/s41569-023-00887-x 37237146

[49] Demmer RT, Allison MA, Cai J, Kaplan RC, Desai AA, Hurwitz BE, Newman JC, Shah SJ, Swett K, Talavera GA et al. Association of Impaired Glucose Regulation and insulin resistance with Cardiac structure and function: results from ECHO-SOL (echocardiographic study of latinos). Circ Cardiovasc Imaging 2016, 9(10). 10.1161/CIRCIMAGING.116.005032 PMC5111817 27729362

[50] Markus MRP, Rospleszcz S, Ittermann T, Baumeister SE, Schipf S, Siewert-Markus U, Lorbeer R, Storz C, Ptushkina V, Peters A (2019). Glucose and insulin levels are associated with arterial stiffness and concentric remodeling of the heart. Cardiovasc Diabetol.

[51] Ai S, Wang X, Wang S, Zhao Y, Guo S, Li G, Chen Z, Lin F, Guo S, Li Y (2022). Effects of glycemic traits on left ventricular structure and function: a mendelian randomization study. Cardiovasc Diabetol.

[52] Cerqueira MD, Weissman NJ, Dilsizian V, Jacobs AK, Kaul S, Laskey WK, Pennell DJ, Rumberger JA, Ryan T, Verani MS (2002). Standardized myocardial segmentation and nomenclature for tomographic imaging of the heart. A statement for healthcare professionals from the Cardiac Imaging Committee of the Council on Clinical Cardiology of the American Heart Association. Circulation.

[53] Ritchie RH, Abel ED (2020). Basic mechanisms of Diabetic Heart Disease. Circ Res.

[54] Litvinukova M, Talavera-Lopez C, Maatz H, Reichart D, Worth CL, Lindberg EL, Kanda M, Polanski K, Heinig M, Lee M (2020). Cells of the adult human heart. Nature.

[55] Kanemaru K, Cranley J, Muraro D, Miranda AMA, Ho SY, Wilbrey-Clark A, Patrick Pett J, Polanski K, Richardson L, Litvinukova M (2023). Spatially resolved multiomics of human cardiac niches. Nature.

[56] Castagno D, Baird-Gunning J, Jhund PS, Biondi-Zoccai G, MacDonald MR, Petrie MC, Gaita F, McMurray JJ (2011). Intensive glycemic control has no impact on the risk of Heart Failure in type 2 diabetic patients: evidence from a 37,229 patient meta-analysis. Am Heart J.

[57] Udell JA, Cavender MA, Bhatt DL, Chatterjee S, Farkouh ME, Scirica BM (2015). Glucose-lowering Drugs or strategies and cardiovascular outcomes in patients with or at risk for type 2 Diabetes: a meta-analysis of randomised controlled trials. Lancet Diabetes Endocrinol.

[58] Stratton IM, Adler AI, Neil HA, Matthews DR, Manley SE, Cull CA, Hadden D, Turner RC, Holman RR (2000). Association of glycaemia with macrovascular and microvascular Complications of type 2 Diabetes (UKPDS 35): prospective observational study. BMJ.

[59] Lind M, Bounias I, Olsson M, Gudbjornsdottir S, Svensson AM, Rosengren A (2011). Glycaemic control and incidence of Heart Failure in 20,985 patients with type 1 Diabetes: an observational study. Lancet.

[60] Usman MS, Siddiqi TJ, Anker SD, Bakris GL, Bhatt DL, Filippatos G, Fonarow GC, Greene SJ, Januzzi JL, Khan MS (2023). Effect of SGLT2 inhibitors on Cardiovascular outcomes across various patient populations. J Am Coll Cardiol.

[61] Ussher JR, Drucker DJ (2023). Glucagon-like peptide 1 receptor agonists: cardiovascular benefits and mechanisms of action. Nat Rev Cardiol.

[62] Burgess S, Thompson SG, Collaboration CCG (2011). Avoiding bias from weak instruments in mendelian randomization studies. Int J Epidemiol.

